# Layering hyperintensity in T1-weighted magnetic resonance imaging predicts gallbladder sludge: a retrospective cohort and diagnostic accuracy study in patients with significant liver disease

**DOI:** 10.1007/s00261-024-04756-0

**Published:** 2025-02-05

**Authors:** Rosa Alba Pugliesi, Timo Siepmann, Daniel P. O. Kaiser

**Affiliations:** 1 University of Palermo, Palermo, Italy; 2 Division of Health Care Sciences Center for Clinical Research and Management Education Dresden, Dresden International University, Dresden, Germany; 3 Department of Neurology, Faculty of Medicine and University Hospital Carl Gustav Carus, TUD Dresden University of Technology, Dresden, Germany; 4 Institute of Neuroradiology, Faculty of Medicine and University Hospital Carl Gustav Carus, TUD Dresden University of Technology, Dresden, Germany

**Keywords:** Gallbladder sludge, Ultrasound (US), Magnetic resonance imaging (MRI), T1w hyperintensity

## Abstract

**Background:**

Layering hyperintensity in the gallbladder is frequently observed on T1-weighted (T1w) magnetic resonance imaging (MRI), but its association with hepatobiliary disorders is not well understood.

**Objective:**

This study aimed to evaluate the prevalence of T1w layering in the gallbladder and its correlation with ultrasound (US) findings and patient characteristics in a cohort with significant liver disease.

**Methods:**

A single-center study from 2015 to 2022 included patients who underwent MRI and abdominal US within one week. Exclusion criteria were poor imaging quality and prior cholecystectomy. MRI findings were correlated with US and analyzed against patient characteristics.

**Results:**

Among 415 patients (mean age 58.3 ± 14.8 years; mean BMI 28.0 ± 4.5 kg/m²), 67% had abnormal liver function tests, with high prevalences of cirrhosis (*n* = 260), transjugular intrahepatic portosystemic shunt (TIPS) (*n* = 233), and choledocholithiasis (*n* = 106). T1w layering was observed in 56% (*n* = 232) and associated with higher BMI (*p* = 0.001) and with cholecystolithiasis (*p* < 0.001), but not with age, sex, or liver disease indicators. T1w layering was predictive of gallbladder sludge on US (odds ratio 17.2, 95% confidence interval 9.87–31.44, *p* < 0.001), with a sensitivity of 92.7% but moderate specificity (57.9%).

**Conclusion:**

T1w layering on MRI strongly predicts gallbladder sludge detected on US and is associated with increased BMI in this cohort of patients with liver disease. However, the moderate specificity requires cautious interpretation, and our findings suggest that T1w layering may serve as a complementary diagnostic tool.

## Introduction

On T1-weighted (T1w) magnetic resonance imaging (MRI) of the abdomen, hyperintensity is often seen in the gallbladder. The signal intensity of bile in the gallbladder and bile duct is variable and depends on the concentration of water, cholesterol, and bile salts. In the non-fasting state, bile usually appears predominantly bright on T2-weighted (T2w) images and hypointense on T1w images, similar to free water [1]. A high-intensity signal from bile on T1w images can be seen in concentrated bile, sludge, stones, or hemobilia [1]. Biliary sludge, a viscous gel-like substance in the gallbladder composed of bile components, can lead to complications such as inflammation and gallstones [2, 3]. Incidental detection of sludge occurs through imaging techniques like MRI or ultrasound (US) [1]. Gallbladder sludge on US refers to a collection of fine, particulate matter composed of cholesterol crystals, calcium bilirubinate granules, and mucin that accumulates in bile [3]. It appears as low-level echoes within the gallbladder without acoustic shadowing, typically layering in dependent areas due to its semi-solid nature [4].

Sludge tends to shift with changes in patient position and can be distinguished from gallstones, which are more echogenic and cast acoustic shadows [5]. In some cases, sludge can be difficult to distinguish from highly concentrated bile or other normal physiological layers, especially after prolonged fasting (NPO) [6]. This is why some cases of “sludge” may be physiological layering, leading to the suggestion that its interpretation should be more cautious or refined [7].

Treatment is typically unnecessary for asymptomatic patients, but complications or symptoms may necessitate cholecystectomy [8, 9]. It is therefore important to distinguish between gallbladder sludge and hyperconcentrated bile that contributes to gallstone formation [10, 11]. Differentiating between the two is challenging, especially on MRI scans, requiring expertise [12], and the presence of a fluid-fluid level on MRI as seen layering T1w hyperintensity does not necessarily indicate sludge [13].

**Fig. 1:**
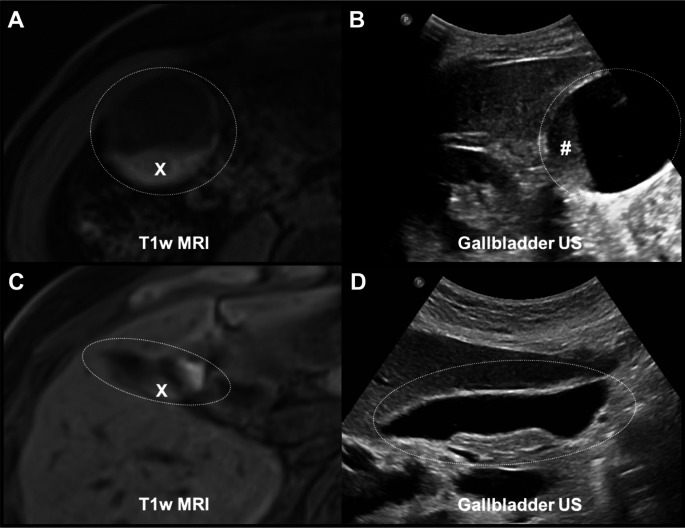
Examples of MRI findings in patients with confirmed and unconfirmed gallbladder sludge. Panel A: T1-weighted (T1w) MRI showing pronounced hyperintensity layering (x), confirmed as sludge (#) by ultrasound (US) in Panel B. Panel C: T1w MRI with light hyperintensity layering (x), suggestive of sludge but not confirmed by the US in Panel D. The gallbladder is delineated with a dotted line

Our analysis focused on evaluating the association between T1w layering on MRI and the presence of sludge as detected by ultrasound. We hypothesized that T1w layering would be a strong predictor of gallbladder sludge (Fig. 1). Additionally, we aimed to explore whether patient characteristics, such as body mass index (BMI), age, and hepatopancreatic disorders, influenced this relationship. Given the high prevalence of liver disease in our cohort, we also sought to explore whether this could affect the generalizability of our findings to a broader, healthier population.

## Methods

### Patients

We retrospectively analyzed patients who underwent an MRI of the abdomen between 2015 and 2022 in an outpatient setting. Only patients who had US within one week were included. The imaging indications, including significant liver conditions such as cirrhosis, portal hypertension, and biliary obstruction, were extracted from the radiology information system. Exclusion criteria included poor imaging quality and previous cholecystectomy or bile duct stents. We also performed a focused subgroup analysis involving patients who underwent the transjugular intrahepatic portosystemic shunt (TIPS) procedure. The study was approved by the Institutional Review Board (IRB number STU00214017), and written informed consent was waived as it was a retrospective study, aligning with institutional guidelines on non-invasive studies involving anonymized data.

### Imaging

We used a 3 Tesla MRI scanner (Siemens MAGNETOM Prisma, Siemens, Erlangen, Germany) equipped with a dedicated 18-channel body matrix coil. For liver or bile system MRI, we used standardized acquisition protocols, including T1-weighted, (fat-suppressed) T2-weighted, and diffusion-weighted imaging, with additional fat-saturated T1-weighted sequences and dedicated contrast media if needed. The patient was prepared by fasting for 4 h prior to the examination, and Buscopan (scopolamine butylbromide) was administered to reduce bowel peristalsis and associated motion artifacts. The T1w, T2w, and fat-suppressed T2w images were acquired with the following parameters: TR = 400–550 ms, TE = 1.5–2.5 ms, FOV = 300–400 mm, and matrix size = 256 × 256. The T2w images were acquired with a slice thickness of 5 mm and an interslice gap of 1 mm, while the fat-suppressed T2w images were acquired with a slice thickness of 5 mm and an interslice gap of 0.5 mm. The T1w images were acquired with a slice thickness of 5 mm and an interslice gap of 0.5 mm.

For gallbladder ultrasound, we used an ultrasound machine (Siemens Sequoia, Siemens, Erlangen, Germany) equipped with curved (1–5 MHz and 3–9 MHz) and linear (4–10 MHz) array transducers. The choice of the transducer was determined by the physicians who conducted the procedure and based on individual patient characteristics, ensuring optimal imaging results. The patient was prepared by fasting for 4 h prior to the examination and the US settings were standardized to maximize sensitivity to bile sludge and gallstones, and measurements were taken in multiple patient positions to differentiate mobile sludge from gallstones.

For this study, the prospectively acquired MRI and US studies were reanalyzed, with diagnostic discrepancies resolved by consensus between two readers. Both readers were board-certified radiologists with expertise in hepatobiliary imaging, and any discordances in findings were resolved through additional imaging review.

### Statistical analysis

Continuous variables are presented as mean and standard deviation (SD), and categorical variables as absolute and relative frequencies. Comparisons between groups were performed using the χ2-test and Student’s t-test. Odds ratios (ORs) were derived from univariate logistic regression modeling. Data were analyzed with IBM SPSS Statistics for Windows, Version 25.0 (IBM Corp., Armonk, NY).

## Results

### Patient characteristics

A total of 415 patients (57.1% males) with a mean age of 58.3 ± 14.8 years and a mean BMI of 28.0 ± 4.5 kg/m² were included (Table 1). The primary indications for imaging were liver cirrhosis, TIPS, and choledocholithiasis. Elevated liver enzymes were observed in 218 patients (Table 1).

**Table 1:** Characteristics of the patient population

Characteristic	*N* (%) or Mean ± SD
All Patients	415 (100%)
Male Sex	237 (57.1%)
Age (years)	58.3 ± 14.8
Body Mass Index (kg/m²)	28.0 ± 4.5
Cholecystolithiasis	106 (25.5%)
TIPS	233 (56.2%)
Liver Cirrhosis	260 (64.6%)
Elevated Liver Enzymes	218 (52.5%)
Ultrasound Findings	
Gallstones	99 (24%)
Sludge	224 (53.9%)
T1w MRI Findings	
Gallbladder Layering	232 (56%)
Gallstones	94 (22.6%)

Patient characteristics. TIPS: transjugular intrahepatic portosystemic shunt, T1w: T1-weighted, MRI: magnetic resonance imaging

### Imaging findings

In our MRI observations, we noted layering hyperintensity in the gallbladder, particularly in T1w imaging, with a prevalence of 56%. Moreover, the combination of T1w and T2w layering was observed in 36% of patients. Patient characteristics were compared between those with and without T1w layering (Table 2). It is noteworthy that T1w and T2w images provided distinct information, with T2w images predominantly showing a bright bile appearance and T1w images appearing hypointense. We detected gallstones in 22.6% of patients based on T1w images.

**Table 2:** Comparison of patient characteristics by T1-weighted (T1w) layering for Sludge Detection. Elevated liver enzymes: AST, ALT

Characteristic	Total Patients (*N* = 415)	T1w Layering (*N* = 232)	No T1w Layering (*N* = 183)	*P*-value
Male Sex, n, % (95% CI)	237, 57.1 (53.6–60.6)	126, 54 (48.7–59.3)	111, 61 (55.3–66.7)	0.195
Age (years) Mean ± SD (95% CI)	58.3 ± 14.8 (57.4–59.2)	58.2 ± 14.9 (57.4–59.2)	58.3 ± 14.9 (57.0–59.4)	0.509
Body Mass Index (kg/m²), mean ± SD (95% CI)	28.0 ± 4.5 (27.57–28.47)	28.0 ± 4.5 (27.57–28.47)	28.0 ± 4.5 (27.55–28.51)	0.001
Cholecystolithiasis, n, % (95% CI)	106, 25.5 (22.1–28.9)	100, 43.1 (38.5–47.7)	6, 3.3 (0.5–6.1)	< 0.001
Liver Cirrhosis, n, % (95% CI)	260, 64.6 (60.9–68.3)	148, 63.8 (58.9–68.7)	112, 61.2 (55.3–67.1)	0.588
Elevated Liver Enzymes, n, % (95% CI)	218, 52.5 (48.6–56.4)	125, 53.9 (49.6–58.2)	93, 50.8 (45.6–55.9)	0.535
Elevated total bilirubin, n, % (95% CI	210, 50.6 (46.6–54.6)	116, 50 (45.1–54.9)	94, 51.4 (45.3–57.5)	0.782

Using ultrasound, gallbladder sludge was detected in 54% of patients, and gallstones were detected in 24% of patients.

### Association of T1w layering and sludge

Our analysis focused on evaluating the association between T1w layering on MRI and the presence of sludge as detected by ultrasound. The diagnostic performance of T1w layering in predicting sludge, as detected by the US, showed a sensitivity of 92.7% and a specificity of 57.9%. Univariate logistic regression analysis revealed a strong predictive relationship, with T1w layering on MRI being significantly associated with the presence of sludge on ultrasound, as indicated by an odds ratio of 17.2 (95% confidence interval [CI]: 9.87–31.44; *p* < 0.001).

For a thorough assessment of diagnostic performance, we calculated the positive predictive value (PPV), which was 68.7%, and the negative predictive value (NPV), which was 88.7%. The model achieved an overall accuracy of 77.3%, and the F1 score, which balances precision and recall, was 82.1%.

**Fig. 2:**
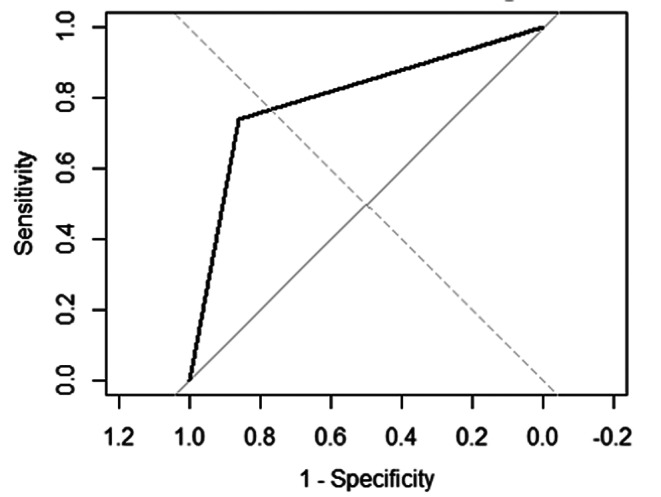
Receiver Operating Characteristic (ROC) curve demonstrating that T1-weighted MRI hyperintensity has strong diagnostic performance for detecting sludge, with an Area Under the Curve (AUC) of 0.956. The optimal cut-off point, closest to the top-left corner, maximizes both sensitivity and specificity

The Receiver Operating Characteristic (ROC) curve analysis demonstrated robust diagnostic performance for MRI T1w layering, with an Area Under the Curve (AUC) of 0.956 (Fig. 2).

### Association of Baseline Characteristics with T1w hyperintensity layering

We explored the association of baseline characteristics with T1w layering, revealing that increased BMI and cholecystolithiasis were the only factors significantly associated with T1w hyperintensity layering on MRI (*p* = 0.001 for BMI and *p* < 0.001 for cholecystolithiasis). No other variables were significantly correlated with layering.

In a focused subgroup analysis involving patients who underwent the TIPS procedure (*n* = 233), a notably higher prevalence of sludge was identified, with 65% confirmed to have sludge in their gallbladders through ultrasound.

## Discussion

In this retrospective cohort and diagnostic accuracy study, we evaluated the clinical significance of T1w hyperintensity layering in the gallbladder and its predictive value for ultrasound-detected gallbladder sludge. Among the 415 patients with an established indication for abdominal imaging, T1w hyperintensity layering was observed in 56% of cases, with 54% of these patients demonstrating sludge on US. Notably, 92.7% of individuals with T1w layering were confirmed to have sludge in the US, underscoring a strong predictive association. These findings are consistent with previous reports [13] and reinforce the clinical relevance of T1w layering as a diagnostic marker.

T1w hyperintensity layering has been previously described in the literature, often attributed to the accumulation of mucus or proteinaceous material within the gallbladder, which may progress to sludge and eventually gallstone formation [12]. This phenomenon has been hypothesized as an early imaging biomarker for gallbladder pathology, offering the potential for timely clinical intervention [14]. While recent advancements in MRI techniques have enhanced the characterization of hepatobiliary disorders, the specific clinical implications of T1w layering remain uncertain [15]. Previous studies have suggested its association with chronic cholecystitis; however, others have struggled to link it to specific clinical symptoms, highlighting a gap that this study seeks to address [13, 16].

The findings of this study may contribute to the understanding of gallbladder pathology in patients with significant liver disease. Our results demonstrated that the presence of T1w layering detects gallbladder sludge with high sensitivity (92.7%). However, the moderate specificity (57.9%) necessitates cautious interpretation in clinical practice, as it may lead to false positives. This limitation highlights the importance of using T1w imaging in conjunction with other diagnostic modalities, such as ultrasound or T2-weighted imaging, to improve accuracy and reduce misdiagnosis. Previous studies have emphasized this multimodal approach as a strategy to enhance diagnostic reliability [17].

The layering phenomenon is thought to result from the accumulation of mucus or proteinaceous material, which can lead to sludge and ultimately gallstone formation. This suggests that T1w hyperintensity layering may serve as an early imaging biomarker for gallbladder pathology, potentially allowing for earlier clinical intervention. Comparatively, studies have shown varying results regarding the prevalence of sludge in liver disease patients, with some reporting a higher incidence in cirrhotic populations [10, 11]. It was highlighted that imaging features in hepatic and gallbladder pathologies might overlap, necessitating careful differentiation to avoid diagnostic inaccuracies [18]. In our cohort T1w layering was associated with the presence of sludge, thereby filling a gap in existing literature regarding imaging characteristics in this demographic. Our cohort predominantly consisted of patients with advanced liver disease, including conditions such as cirrhosis, portal hypertension, transjugular intrahepatic portosystemic shunt (TIPS) placement, and elevated liver function tests. This skewness toward significant liver pathology limits the generalizability of our findings to broader populations. Patients with hepatic dysfunction often exhibit alterations in the biliary milieu, which may predispose them to higher rates of gallbladder abnormalities, including sludge. Although these associations are supported by previous studies [16, 19], future research should prioritize diverse cohorts to evaluate whether similar correlations hold across varied clinical settings.

In addition to cholecystolithiasis (*p* < 0.001), elevated body mass index (BMI) was significantly associated with T1w layering in gallbladder sludge (*p* = 0.003). This finding may reflect altered bile composition, impaired gallbladder motility, and hormonal changes commonly observed in individuals with obesity. These findings are consistent with prior research [20, 21] and underscore the interplay between metabolic and biliary factors. However, other potential contributors, such as dietary habits and genetic predispositions, were not examined in this study. Future investigations should incorporate these variables to provide a more comprehensive understanding of biliary pathology [22, 23].

Interestingly, our study did not identify sex or age as significant predictors of gallbladder sludge, despite their well-established roles in gallstone formation [24]. This divergence from existing literature may reflect differences in the underlying mechanisms driving sludge versus gallstone development. The lack of association with markers of liver disease, such as cirrhosis, also warrants further investigation to delineate the specific pathophysiological processes influencing T1w layering.

The high prevalence of T1w hyperintensity layering and sludge in our cohort may, in part, be attributed to physiological bile hyperconcentration during non-per-oral (NPO) states. This finding aligns with previous studies suggesting that T1w layering may not always indicate pathology but instead reflect normal gallbladder dynamics [25, 26]. Distinguishing between physiological and pathological layering is critical to improving diagnostic specificity and preventing overdiagnosis. Future studies should focus on refining criteria to differentiate these phenomena.

Gallstone detection in our study was consistent with previously reported prevalence rates, with 24% of patients demonstrating gallstones on imaging. T1w imaging revealed also high sensitivity imaging for gallstone detection showing its valuable diagnostic role in settings where cost and accessibility are limiting factors [27, 28]. This reinforces the complementary role of T1w MRI alongside traditional imaging modalities, particularly in resource-constrained environments.

With an overall diagnostic accuracy of 77.3% and an area under the receiver operating characteristic (ROC) curve of 0.956, T1w layering emerges as a promising predictive marker, particularly for ruling out the absence of sludge. However, the moderate specificity necessitates a cautious approach, emphasizing the need for integration with clinical and laboratory data to enhance diagnostic confidence [29–31].

Despite the valuable findings of this study, several limitations must be acknowledged. As a single-center, retrospective study, the results are susceptible to selection bias and may not be generalizable to other populations. The lack of standardized follow-up and the potential for misinterpretation of physiologic bile hyperconcentration as pathologic cholestasis further limit the applicability of our results. Nevertheless, the homogeneity of data collection and analysis strengthens the internal validity of our findings.

## Conclusion

In conclusion, our study contributes to the growing body of knowledge regarding T1-weighted hyperintensity layering in the gallbladder as a potential indicator of gallbladder sludge, highlighting its diagnostic relevance. While the strong association between T1-weighted imaging and sludge detection underscores its potential utility, the moderate specificity suggests that caution is necessary, particularly in patients without significant liver disease. The findings must be interpreted with consideration of the high prevalence of liver disease in the study cohort, which may limit the broader applicability of the results.

To maximize the clinical utility of T1-weighted imaging, it should be integrated into a broader diagnostic framework, rather than relying on it as a stand-alone marker. Future research should focus on multicenter studies with a more diverse patient population to further investigate the diagnostic capabilities of T1-weighted layering in different clinical contexts. Additionally, exploring the relationship between MRI and ultrasound findings, along with their potential implications for biliary complications, will be crucial in refining the clinical application of this imaging technique. Ultimately, a more comprehensive understanding of T1-weighted hyperintensity layering will aid in enhancing the diagnosis and management of gallbladder disease.

## Data Availability

The data that support the findings of this study are available from the corresponding author upon reasonable request.
